# LDL-c Lowering, Ischemic Stroke and Small Vessel Disease Brain Imaging Biomarkers: A Mendelian Randomization Study

**DOI:** 10.1161/STROKEAHA.123.045297

**Published:** 2024-04-04

**Authors:** Marie-Joe Dib, Loukas Zagkos, Devendra Meena, Stephen Burgess, Julio A. Chirinos, Dipender Gill

**Affiliations:** 1 Division of Cardiovascular Medicine, Hospital of the University of Pennsylvania, Philadelphia PA; 2 Department of Epidemiology and Biostatistics, School of Public Health, Imperial College London, UK; 3 MRC Integrative Epidemiology Unit, University of Bristol, Bristol, UK

**Keywords:** stroke, perivascular spaces, white matter hyperintensity volume, small vessel disease, Mendelian randomization, drug target

## Abstract

**Background:**

The effects of lipid-lowering drug targets on different ischemic stroke (IS) subtypes are not fully understood. We aimed to explore the mechanisms by which lipid-lowering drug targets differentially affect the risk of IS subtypes and their underlying pathophysiology.

**Methods:**

Using a two-sample Mendelian randomization (MR) approach, we assessed the effects of genetically-proxied low-density lipoprotein cholesterol (LDL-c) and 3 clinically approved LDL-lowering drugs (3-hydroxy-3-methylglutaryl-CoA reductase, HMGCR; proprotein convertase subtilisin/kexin type 9, PCSK9 and Niemann-Pick C1-Like 1, NPC1L1) on stroke subtypes and brain-imaging biomarkers associated with small vessel disease (SVS), including white matter hyperintensity volume (WMHV) and perivascular spaces (PVS).

**Results:**

In genome-wide MR analyses, lower genetically predicted LDL-c was significantly associated with a reduced risk of any stroke (AS), IS and large artery stroke (LAS), supporting previous findings. Significant associations between genetically predicted LDL-c and cardioembolic stroke (CES), SVS and biomarkers PVS and WMHV were not identified in this study. In drug-target MR analysis, genetically-proxied reduced LDL-c through NPC1L1 inhibition was associated with lower odds of PVS [Odds ratio (OR) per 1 mg/dL decrease = 0.79; 95% confidence interval (CI) = 0.67-0.93], and with lower odds of SVS [OR= 0.29, 0.10-0.85].

**Conclusions:**

This study provides supporting evidence of a potentially protective effect of LDL-c lowering through NPC1L1 inhibition on PVS and SVS risk, highlighting novel therapeutic targets for SVS.

## Graphical abstract

**Figure:**
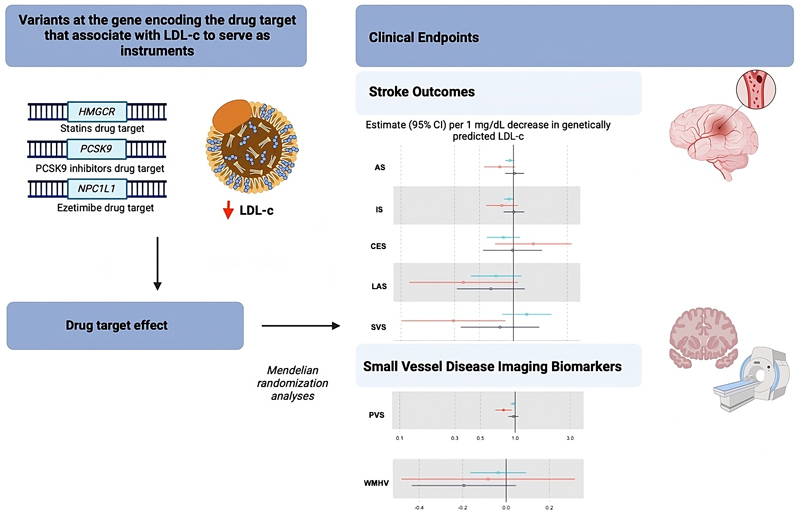
Graphical Abstract

## Nonstandard abbreviations and acronyms

AS: any stroke
CI: confidence interval
HMGCR: 3-hydroxy-3-methylglutaryl-CoA reductase
IS: ischaemic stroke
LAS: large artery stroke
LDL-c: low density lipoprotein cholesterol
MR: Mendelian randomization
NPC1L1: Niemann-Pick C1-Like 1
OR: odds ratio
PCSK9: proprotein convertase subtilisin/kexin type 9
PVS: perivascular space
SVS: small vessel stroke
WMH: white matter hyperintensity

## Introduction

Ischemic stroke (IS) is a leading cause of death, with the main aetiological subtypes being large-artery stroke (LAS), cardioembolic stroke (CES), and small-vessel stroke (SVS). LDL-c lowering drug targets are efficacious for reducing atherosclerotic cardiovascular disease (ASCVD) risk, but their beneficial effect across IS subtypes and underlying mechanisms are not well understood. Additionally, up to 30% of IS are cryptogenic, characterized by a lack of insight into their underlying mechanism, leading to uncertainty in therapeutic choices^1^. Heterogeneity within IS subtypes requires improved distinction for more effective disease management strategies.

Emerging brain imaging biomarkers may offer novel insight towards elucidating the underlying pathophysiology of IS subtypes, and could thus guide risk stratification for optimized treatment approaches. Mendelian randomization (MR) is a statistical method that employs genetic variation as an instrumental variable to assess the causal relationship between exposures and outcomes of interest. This approach mitigates the risk of confounding and reverse causality, that are commonly found in traditional epidemiological studies. Here, we leverage the largest genome-wide association studies (GWAS) of IS subtypes to date (GIGASTROKE Consortium, N_cases_= 110,182)^2^, and distinct magnetic resonance brain-imaging biomarkers data to explore how distinct lipid-lowering drug targets differentially affect risk of various IS subtypes and their underlying pathophysiology.

## Methods

We investigated the associations between genetically predicted LDL-c, IS subtypes, and brain biomarkers (perivascular spaces (PVS) and white matter hyperintensity (WMH) volumes) in a two-sample MR design. We then applied drug-target MR using genetic instruments associated with 3 druggable gene targets (*HMGCR, PCSK9*, and *NPC1L1*) to genetically proxy and evaluate their lifelong impact on outcomes of interest. Assumptions of MR pertain to the validity of genetic variants employed as instrumental variables. Genetic variants should (1) strongly predict the exposure under study, (2) exhibit associations with the outcome only through the exposure, (3) not be associated with confounders of the exposure-outcome association. The main analysis was conducted using the random-effects inverse-variance weighted (IVW). We implemented the weighted median estimator (WM), MR-Egger, and the contamination-mixture (Conmix) methods for sensitivity analyses. Details of the methodology used is available in the Online Supplement. Summary level data were publicly available, and all studies have been approved by corresponding ethical review committees. This study is reported using the Strengthening the Reporting of Observational Studies in Epidemiology-Mendelian randomization (STROBE-MR) guidelines (https://www.strobe-mr.org) (Online Supplement).

## Results

The MR estimate results of the primary analyses are shown in Figure S1 and Table S1. Analyses indicated adequate instrument strength (mean F statistic>10). We found that lower genetically predicted LDL-c was associated with reduced odds of LAS, AS and IS after correcting for multiple testing. The estimates computed through the use of our selected genetic instruments were heterogeneous for IS and LAS (Cochran’s Q *P*<0.001). For all outcomes under consideration, the Egger intercept test did not support evidence for pleiotropy (*P* > 0.05).

**Figure 1:**
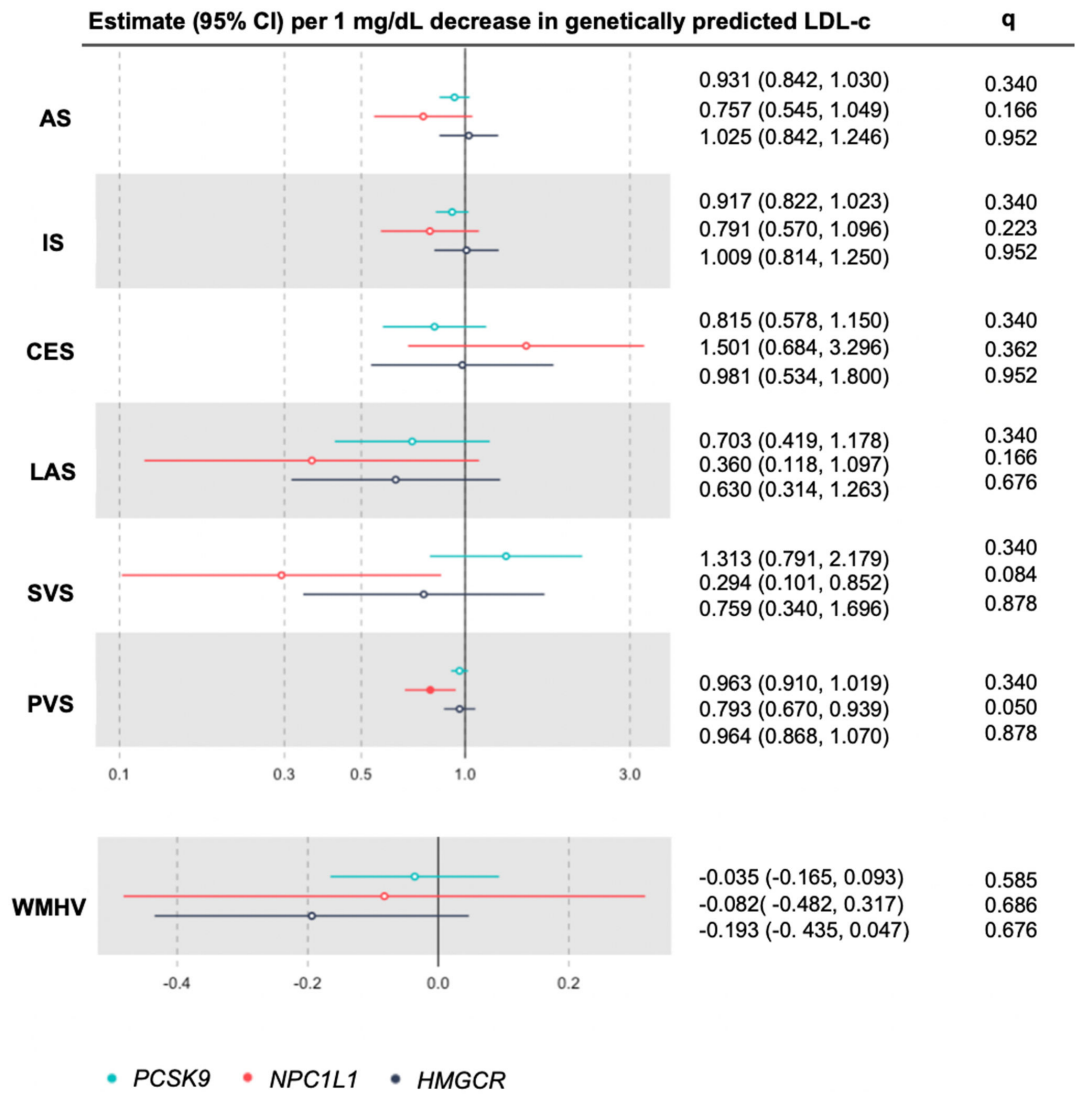
Mendelian randomization estimates for associations between a 1 mg/dL decrease in genetically predicted LDL-c, stroke risk, and brain-imaging biomarkers through the inhibition of 3 drug targets (A) HMGCR, (B) PCSK9, (C) NPC1L1. Estimates are expressed in odds ratios when outcomes are binary, and in betas when outcomes are continuous. Shapes that are colour filled indicate *q* values < 0.05. We report FDR corrected *P* values (*q* values). AS, any stroke; CES, cardioembolic stroke; Conmix, contamination-mixture; IS, ischaemic stroke; IVW, inverse-variance weighted; LAS, large artery stroke; LDL-c, low density lipoprotein cholesterol; PVS, perivascular space; SVS, small vessel stroke; WM, weighted median; WMH, white matter hyperintensity volumes.

Full results of drug target MR analyses are in Figure 1 and Table S2-S3. Considering the effects of reduced LDL-c levels through genetically-proxied NPC1L1 inhibition on the outcomes under study, MR analyses identified an association with lower risk of PVS after adjusting for multiple testing, and SVS at nominal significance. We did not identify heterogeneous effects among MR tests across all outcomes under study (Cochran’s *P*>0.05, Table S4). Using PhenoScanner, we identified 2 potential pleiotropic variants in *HMGCR* and 3 in *PCSK9* (Table S5). Sensitivity MR estimates excluding these variants did not substantially differ from the main MR estimates (Table S6).

## Discussion

Our findings suggest a potentially beneficial effect of NPC1L1 inhibition on the risk of developing PVS and SVS. We also support previous findings highlighting an association between genetically predicted LDL-c and risk of AS, IS and LAS.

Consistent with our results, prior MR investigations have suggested that genetically predicted LDL-c levels are associated with a higher propensity to LAS, while no significant associations were observed with SVS or CES^3,4^. Our findings emphasize the need to further understand the aetiology of IS subtypes and improve their classification with more specific brain-imaging biomarkers.

Our MR study highlights an association between genetically-proxied LDL-c reduction mediated by NPC1L1 inhibition and a reduction in PVS. A previous MR study reported a reduction in risk of SVS as a consequence of NCP1L1 mediated LDL-c lowering^3^, which we replicate in our study at nominal significance. Together, these findings suggest that the pharmacological targeting of NPC1L1 may offer a novel approach to managing cerebrovascular disease, with potential implications for cognitive health. Subsequent investigations into the role of NPC1L1 inhibition in mitigating the risk of cognitive impairment associated with SVD is warranted, aiming to contribute to the reduction of the global burden of aging-related cognitive decline. Our results thereby provide a genetic basis for exploring NPC1L1 as a potential novel therapeutic target.

Our study brings notable advances to the field, in that it (1) leverages the largest sample size of stroke outcomes to date, and is the first to investigate (2) brain-imaging biomarkers as outcomes, and (3) gene-specific effects of LDL-c modulation on these outcomes through drug targets. Our study also has a number of limitations. The estimates derived from our MR experiments may not be comparable to estimates reported in clinical practice or in RCTs. Our analyses used GWAS resources pertaining to populations of European ancestry, therefore our findings may not be generalizable to other populations. While our study provides valuable insights into the genetic basis of licensed and late-stage lipid-lowering drug targets, it is essential to acknowledge that the broader clinical context involves a myriad of factors that may not be fully accounted for in MR analyses. These include, lifestyle factors, additional cardiovascular risk factors, and various pharmacological interventions and their interactions. The latter are important to consider as drug combinations may exert synergistic effects. Therefore, our findings should be assessed in properly designed RCTs. Lastly, investigating the effects of other lipid-lowering drug targets that are in early stages of drug development may provide further mechanistic insight underpinning the heterogeneity of IS subtypes and should also be the focus of future studies.

In conclusion, our MR study highlighted a significant association between lower genetically predicted LDL-c through NPC1L1 inhibition and a reduced risk of PVS and SVS. These findings are of clinical importance as they shed light on the distinct mechanisms of action of lipid-lowering drugs through effects on brain-imaging biomarker PVS, and add to the evidence supporting a potential beneficial effect of lipid-lowering medication on biomarkers of SVD, and thus potentially also related cognitive function. Further evidence from clinical studies on the effects of NPC1L1 inhibitors on PVS, SVS and cognitive function, is needed to clinically validate our findings.

## Supplementary Material

Graphical Abstract

Graphical Abstract

STROBE checklist

Supplemental Publication Material
