# Supplementary figures and images for: LDL-c Lowering, Ischemic Stroke and Small Vessel Disease Brain Imaging Biomarkers: A Mendelian Randomization Study
Source: Stroke. Author manuscript; Available in PMC 2024 Jun 1. (PMC7615976; doi:10.1161/STROKEAHA.123.045297)

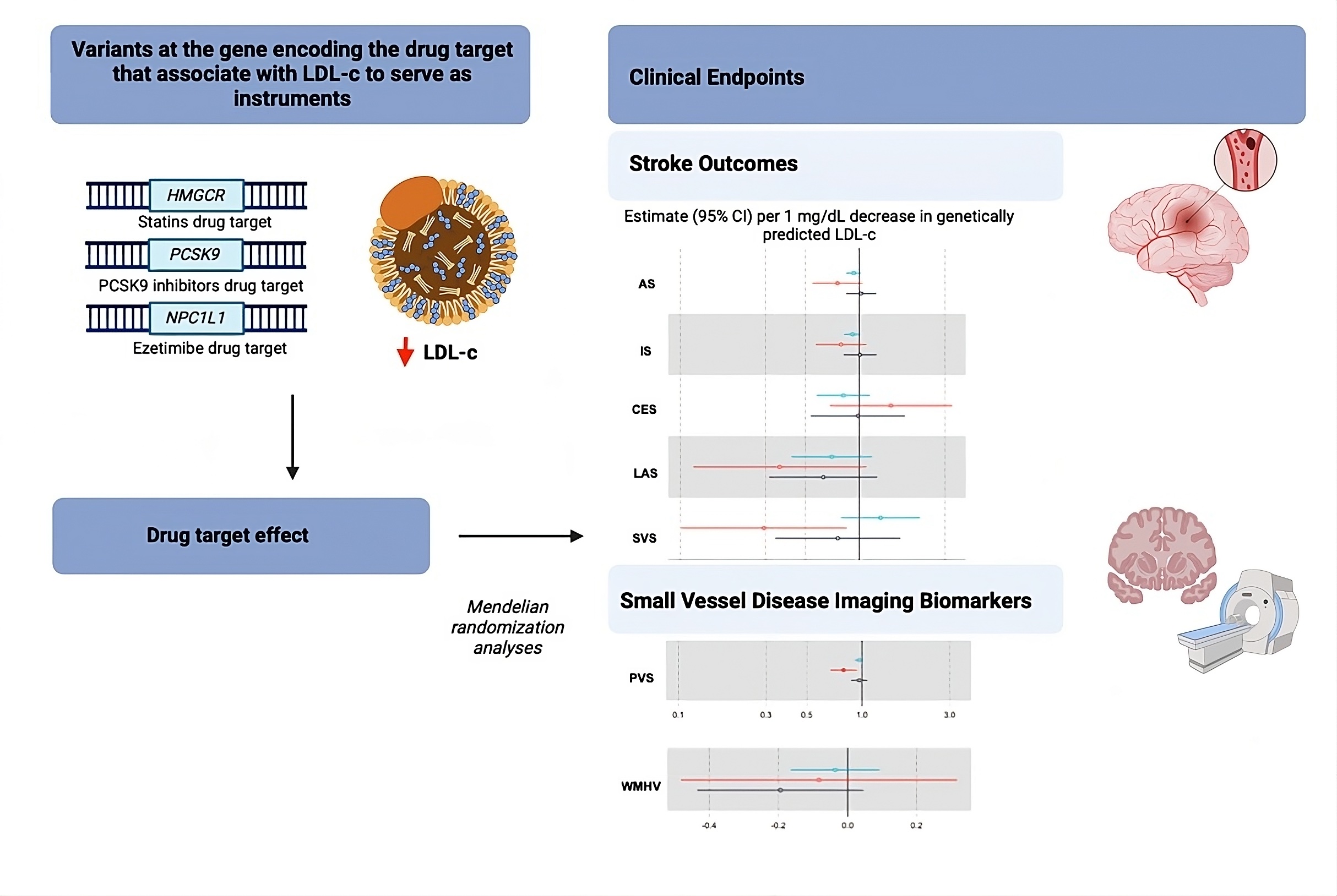

Supplement: Graphical Abstract [file EMS194880-supplement-Graphical_Abstract.jpg]
