# Supplementary material for: LDL-c Lowering, Ischemic Stroke and Small Vessel Disease Brain Imaging Biomarkers: A Mendelian Randomization Study
Source: Stroke. Author manuscript; Available in PMC 2024 Jun 1. (PMC7615976; doi:10.1161/STROKEAHA.123.045297)
Supplement: Supplemental Publication Material [file EMS194880-supplement-Supplemental_Publication_Material.pdf]

Title: LDL-c Lowering, Ischemic Stroke and Small Vessel Disease Brain Imaging Biomarkers: A Mendelian Randomization Study

Marie-Joe Dib, PhD[1,2*], Loukas Zagkos, PhD [3], Devendra Meena, DPhil[3], Stephen Burgess, PhD[4,5], Julio A. Chirinos, MD, PhD[1,2], Dipender Gill, MD, PhD[3]

[1] Division of Cardiovascular Medicine, Hospital of the University of Pennsylvania, Philadelphia PA

[2] University of Pennsylvania Perelman School of Medicine, Philadelphia, PA

[3] Department of Epidemiology and Biostatistics, School of Public Health, Imperial College London, UK.

[4] MRC Integrative Epidemiology Unit, University of Bristol, Bristol, UK.

[5] Department of Public Health and Primary Care, University of Cambridge, Cambridge, UK.

*Corresponding author

Address for correspondence:
Marie-Joe Dib, PhD
Perelman Center for Advanced Medicine.
3400 Civic Center Blvd. Philadelphia, PA. 19104.
Email: marie-joe.dib@pennmedicine.upenn.edu
Twitter: @mariejoedib

<u>Online Supplement</u>

Expanded Methods

Genetic association data

We obtained genetic instruments for LDL-c from participants of European ancestry from the Global Lipid Genetics Consortium (GLGC, N=1,320,016) [5]. LDL-c was measured after >8 hours fasting in most studies nested in the GLGC metaGWAS. Participants are of European ancestry. Genetic associations for stroke and subtypes were obtained from the GIGASTROKE Consortium [2]. Genetic associations for PVS were obtained from a meta-GWAS of 18 cohorts from the UK Biobank, the CHARGE consortium and the BRIDGET initiative (N=40,095). PVS were defined as fluid-filled spaces with a signal of round, ovoid or linear shape with a maximum diameter smaller than 3mm, no hypertensive rim on T2-weighted or FLAIR sequences, and located in areas supplied by perforating arteries (N=40,095)[6]. PVS was considered as a categorical variable, defined as presence or absence of extensive PVS burden (N_{cases}=9,339). Summary statistics for WMH volumes estimates were obtained from volumetric brain MRI-derived phenotypes in the UK Biobank (n=33,224) and are represented in mm^3.[7]

Genetic instrument selection

In univariable MR analyses investigating overall effects of LDL-c, we first identified genome wide significant (P<5 x 10^{-8}) single-nucleotide polymorphisms (SNPs) associated with LDL-c that were also present in the outcome GWAS dataset. Effect alleles in exposure and outcome datasets were harmonized, and the remaining variants were clumped at linkage disequilibrium (LD) r^2<0.001 and a 10-Mb window using the 1000 Genomes Project Phase 3 European LD reference panel to ensure the selection of uncorrelated SNPs. When variants were missing in the outcome GWAS, they were replaced by proxy variants in high LD with

the initial SNP of interest. Instrument strength was assessed by computing the F statistic for each instrument. We report the mean F statistic for the totality of genetic instruments selected for the exposure.

To proxy effects of modifying LDL-c through single genes encoding drug targets for LDL-c lowering therapies, we selected SNPs previously identified as genetic proxies for modulation of *HMGCR* (targeted by statins, gene region (GRCh37/hg19): chr5:74,632,154-74,657,929), *PCSK9* (targeted by PCSK9 inhibitors, chr1:55,505,221-55,530525), and *NPC1L1* (targeted by ezetimibe, chr7:44,552,134-44,580,929). For these drug target MR analyses, we selected SNPs within ±100 kb from each of the target genes (PCSK9, HMGCR, and NPC1L1). For genetic instruments, we considered SNPs associated with LDL-c at a genome-wide significance level ($P < 5.0 \times 10^{-8}$), and clumped these SNPs using a pair-wise LD cut-off of $r^2 < 0.1$. When variants were missing in the outcome GWAS, they were replaced by proxy variants in high LD with the initial SNP, when available. We report the variants used as genetic instruments for each exposure-outcome combination in **Table S1**. To determine the statistical power to detect a true causal effect in Mendelian randomization analyses, we estimate and report the variance in LDL-c explained by each SNP.

Investigation of Genetic Pleiotropy

The robustness of MR estimates may be compromised if the selected genetic instruments have an influence on the considered outcomes of interest through a pleiotropic pathway that is independent of the drug under study. To address this concern in drug-target MR, we utilized the PhenoScanner[8] curated database to investigate whether the selected SNPs were significantly associated (at genome-wide level) with other traits or diseases that may represent pleiotropic pathways. PhenoScanner includes SNP-phenotype associations

identified in analysis of UK Biobank data. SNPs that were identified as having pleiotropic effects were excluded in sensitivity analyses.

Mendelian randomization estimates

The main analysis was conducted using the random-effects inverse-variance weighted (IVW). This method provides a consistent causal estimate if the genetic variants employed as instrumental variables meet the instrumental variable assumptions. MR here aims to weigh the effect of the outcome by a SD increase in LDL-c levels. We conducted a statistical test for heterogeneity using Cochran's Q to test for potential horizontal pleiotropy in the causal effects estimated by each of the genetic proxies for LDL-c. We further implemented methods that are more robust to horizontal pleiotropy, including the weighted median estimator, MR-Egger, and the contamination mixture method. The contamination-mixture method assumes that the MR estimates obtained from valid instruments follow a normal distribution centred on the true causal effect estimate and those derived from invalid instruments follow a normal distribution centred on the null.[9] A likelihood function is then specified and maximized for allocating each instrument variant to one of the two mixture distributions. We considered associations to be statistically significant when *P* values in MR-IVW, MR-WM, and MR-ConMix methods met the significance threshold. MR-Egger was considered supportive when the effect estimate was in the same direction as MR-IVW and the MR-Egger intercept was statistically nonsignificant. For drug target MR, we implemented the MR-IVW as our main method, adjusting for correlation between genetic instruments in the analysis.

We estimated the false discovery rate (FDR) adjusted *P* values (q values) to address testing of multiple correlated phenotypes. A q value < 5% was considered as suggestive of strong evidence of association. Two-sample MR analyses were performed using the TwoSampleMR

package (version 0.5.6) and MendelianRandomization package (version 0.9.0) in R (version 5.12.10; The R Foundation for Statistical Computing, Vienna, Austria).

Figure S1. Associations of 1 mg/dL decrease in genetically predicted LDL cholesterol through any mechanism with stroke outcomes (AS, IS, CES, LAS, SVS) and brain-imaging biomarkers WMH and PVS. Mendelian randomization (MR) inverse variance weighted (IVW), weighted median (WM) and contamination-mixture (Conmix) estimates are expressed in odds ratios and 95% confidence intervals when outcomes are binary, and in betas and 95% confidence intervals when outcomes are continuous. Shapes that are colour filled indicate *q* values<0.05. We report FDR corrected *P* values (q values).

AS, any stroke; CES, cardioembolic stroke; Conmix, contamination-mixture; IS, ischaemic stroke; IVW, inverse-variance weighted; LAS, large artery stroke; LDL-c, low density lipoprotein cholesterol; PVS, perivascular space; SVS, small vessel stroke; WM, weighted median; WMHV, white matter hyperintensity volumes.

Figure S1

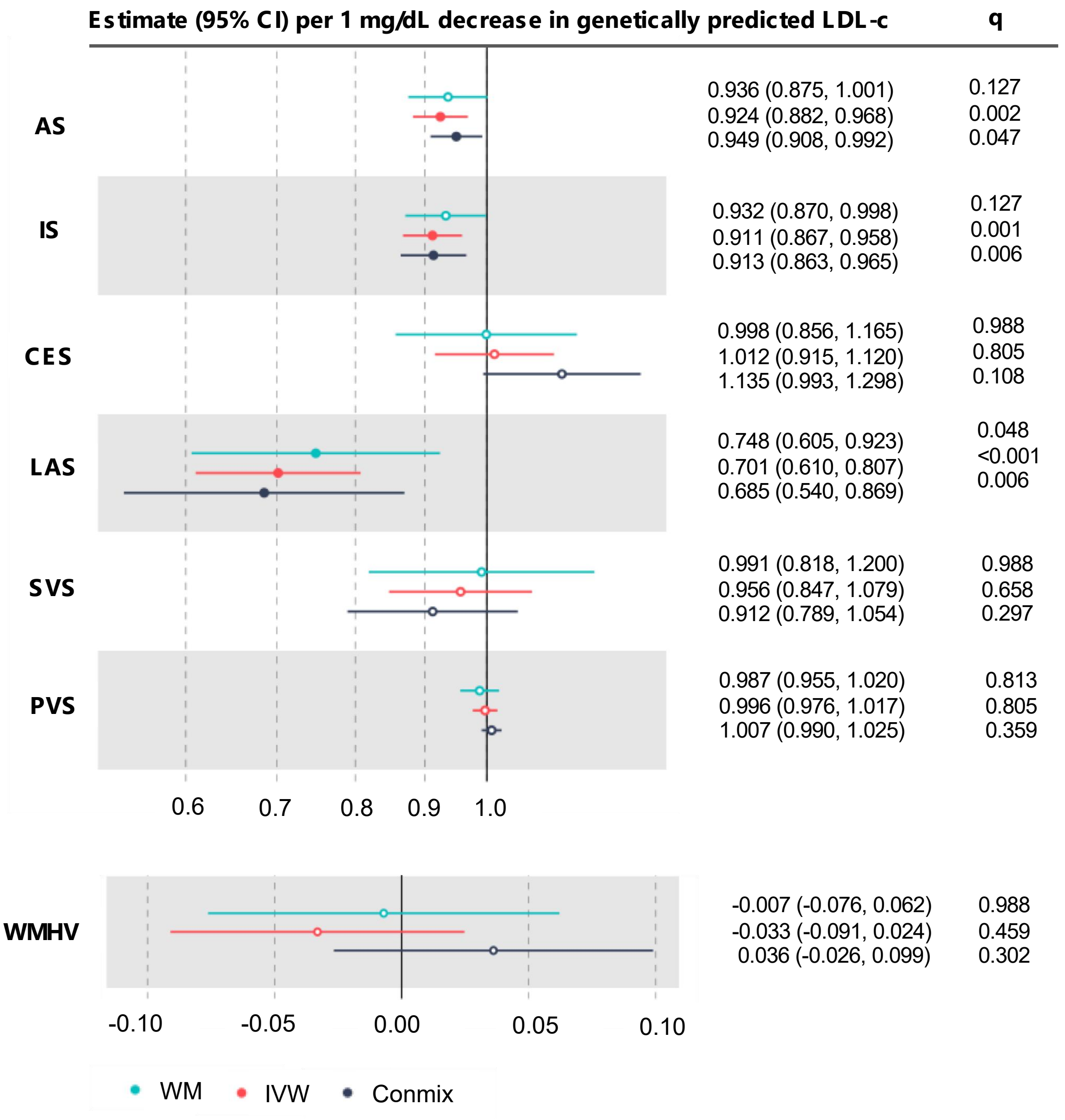

Estimate (95% CI) per 1 mg/dL decrease in genetically predicted LDL-c
q
AS
0.936 (0.875, 1.001) 0.127
0.924 (0.882, 0.968) 0.002
0.949 (0.908, 0.992) 0.047
IS
0.932 (0.870, 0.998) 0.127
0.911 (0.867, 0.958) 0.001
0.913 (0.863, 0.965) 0.006
CES
0.998 (0.856, 1.165) 0.988
1.012 (0.915, 1.120) 0.805
1.135 (0.993, 1.298) 0.108
LAS
0.748 (0.605, 0.923) 0.048
0.701 (0.610, 0.807) <0.001
0.685 (0.540, 0.869) 0.006
SVS
0.991 (0.818, 1.200) 0.988
0.956 (0.847, 1.079) 0.658
0.912 (0.789, 1.054) 0.297
PVS
0.987 (0.955, 1.020) 0.813
0.996 (0.976, 1.017) 0.805
1.007 (0.990, 1.025) 0.359
0.6
0.7
0.8
0.9
1.0
WMHV
-0.007 (-0.076, 0.062) 0.988
-0.033 (-0.091, 0.024) 0.459
0.036 (-0.026, 0.099) 0.302
-0.10
-0.05
0.00
0.05
0.10
WM
IVW
Conmix
